# Case of Nocardia Cyriacigeorgica Infection of the Eye in a Granulomatosis With Polyangiitis Patient

**DOI:** 10.7759/cureus.11178

**Published:** 2020-10-26

**Authors:** Khalid Alshammari, Bushra Al Hothaly, Fahad Alrabiah

**Affiliations:** 1 Medicine, University of Hail College of Medicine, Hail, SAU; 2 Infectious Diseases, King Faisal Specialist Hospital and Research Centre, Riyadh, SAU

**Keywords:** farms, nocardia cyriacigeorgica, granulomatosis with polyangiitis, eye infection, animals

## Abstract

Nocardiosis is an infectious disease caused by a group of organisms that are often found in soil and has a very rare incidence of infecting immunocompromised patients. Granulomatosis with polyangiitis patients are often susceptible to being infected with many atypical organisms such as Nocardia cyriacigeorgica. We present a case of a 35-year-old male who is a known case of granulomatosis with polyangiitis and has a repeated history of farm visits. The patient presented with progressive early morning right eye secretions followed by dryness throughout the day with no history of trauma or allergy of seven months duration. An eye swab for culture and sensitivity showed an isolated Nocardia cyriacigeorgica and was treated by trimethoprim/sulfamethoxazole (Bactrim) for one year but was lost to follow-up. Early detection of Nocardia cyriacigeorgica is crucial in those groups of patients, as it can prevent further complicated outcomes while proper hygiene education is important.

## Introduction

Nocardia are a group of branching, hyphae-like, aerobic, gram-positive, and weak, filamentous acid-fast bacillus with over 50 species being described in the literature. Furthermore, they are members of the Nocardiaceae family and belongs to the suborder of aerobic actinomycetes [1-2]. They are considered opportunistic organisms that are found extensively in soil with the ability to infect individuals in extremely rare incidents, with various subtypes under it, which include but are not limited to Nocardia abscessus,Nocardia cyriacigeorgica*, *Nocardia brasiliensis*, *and Nocardia asteroids[3]. Even though opportunistic organisms have a general tendency to infect immunocompromised individuals more frequently, only one-third of Nocardiosis patients have impaired immunity [4]. Despite the fact that infection in granulomatosis with polyangiitis (GPA) remains one of the most common hospitalization reasons in that group, in the last two decades, the mortality rate of infection in GPA greatly decreased [5].

## Case presentation

A 35-year-old male, working at an administrative job, was a known case of GPA, which was diagnosed in 2012. During the same year, he presented to our hospital with recurrent relentless hemoptysis, cough, and epistaxis and received a second dose of rituximab with no indication of relapse as antineutrophil cytoplasmic antibodies (ANCA) were not elevated. Further investigation was elicited, including chest computed tomography (CT), which showed a large area of consolidation involvement mainly in the upper left lobe of the lung and the lingua, as well as a paranasal sinuses (PNS) CT, which showed mucosa thickening of the maxillary sinus on the left side. The treatment plan included rituximab every six to nine months, along with prednisone 5 mg oral daily and azathioprine 100 mg daily. In 2013, azathioprine and rituximab were replaced by cyclophosphamide 1500 mg due to recurrent relapse, as he received only four doses and then returned to rituximab as the maintenance dose. In December 2015, the patient was complaining of excessive tearing and underwent bilateral Grommet insertion with right endoscopic dacryocystorhinostomy (DCR) without complications arising.

In 2019, the patient presented to the clinic, complaining of progressive early morning right eye secretions followed by dryness throughout the day, with no history of trauma or allergy of seven months duration. On further questioning, the patient confirmed close contact with various animals when he was at his farm, which included camels, cattle, birds, dogs, and horses. Furthermore, there was a history of multiple travels to multiple Asian, European, and Middle Eastern countries, as well as a history of drinking unpasteurized milk. In addition, the patient was a long-time user of traditional eyeliner (i.e., ithmid). Upon physical examination, there was only mild tearing mainly in the right eye and no signs of redness, itching, thickening, scleral discoloration, corneal abrasions, irritation to light, or any discharge from the eye, and a swab was taken. Regarding the swab, the culture showed an isolated Nocardia cyriacigeorgica organism, which was confirmed with a second swab that was sent to Mayo Clinic, and the patient was admitted to the ward. During the hospital course, a multi-investigational approach was done from blood work, which included complete blood count (CBC), C-reactive protein (CRP), erythrocyte sedimentation rate (ESR), serology, renal and bone profile, as well as imaging, which included chest CT, PNS CT, and brain CT.

**Figure 1:**
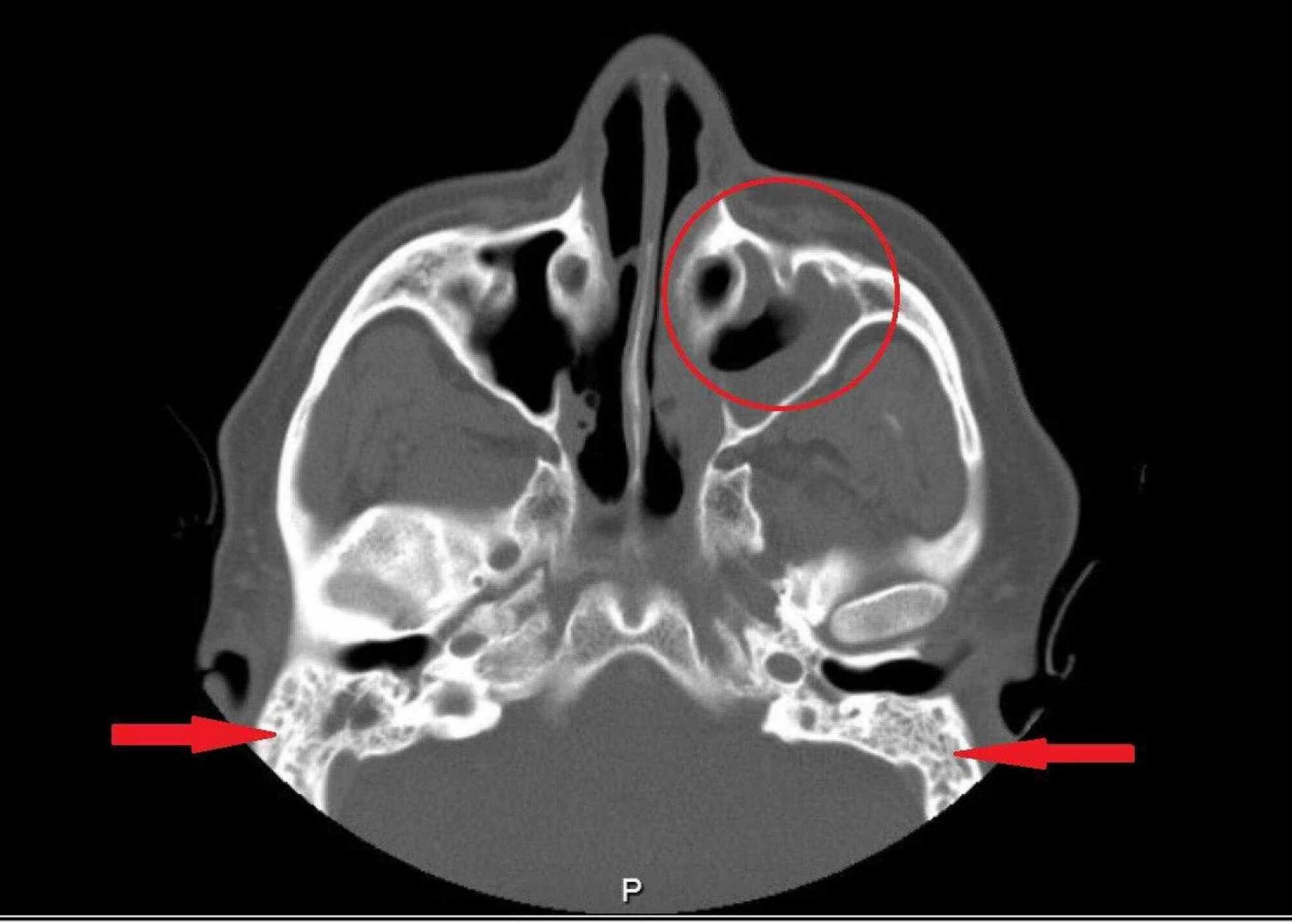
Paranasal sinuses computed tomography demonstrated bilateral otomastoiditis with interval development of severe left maxillary sinus mucosal disease.

Regarding his blood work, CBC showed lymphopenia, which was regular due to the nature of the immunosuppression drugs, normal range of CRP, ESR, and renal and bone profile. On the other hand, brain and orbit CT scan showed complete opacification of the bilateral mastoid air cells and middle ear cavities, new mucosal disease of the paranasal sinuses suggestive of active GPA, and no reported abnormalities in the orbital region (Figure 1).

**Figure 2:**
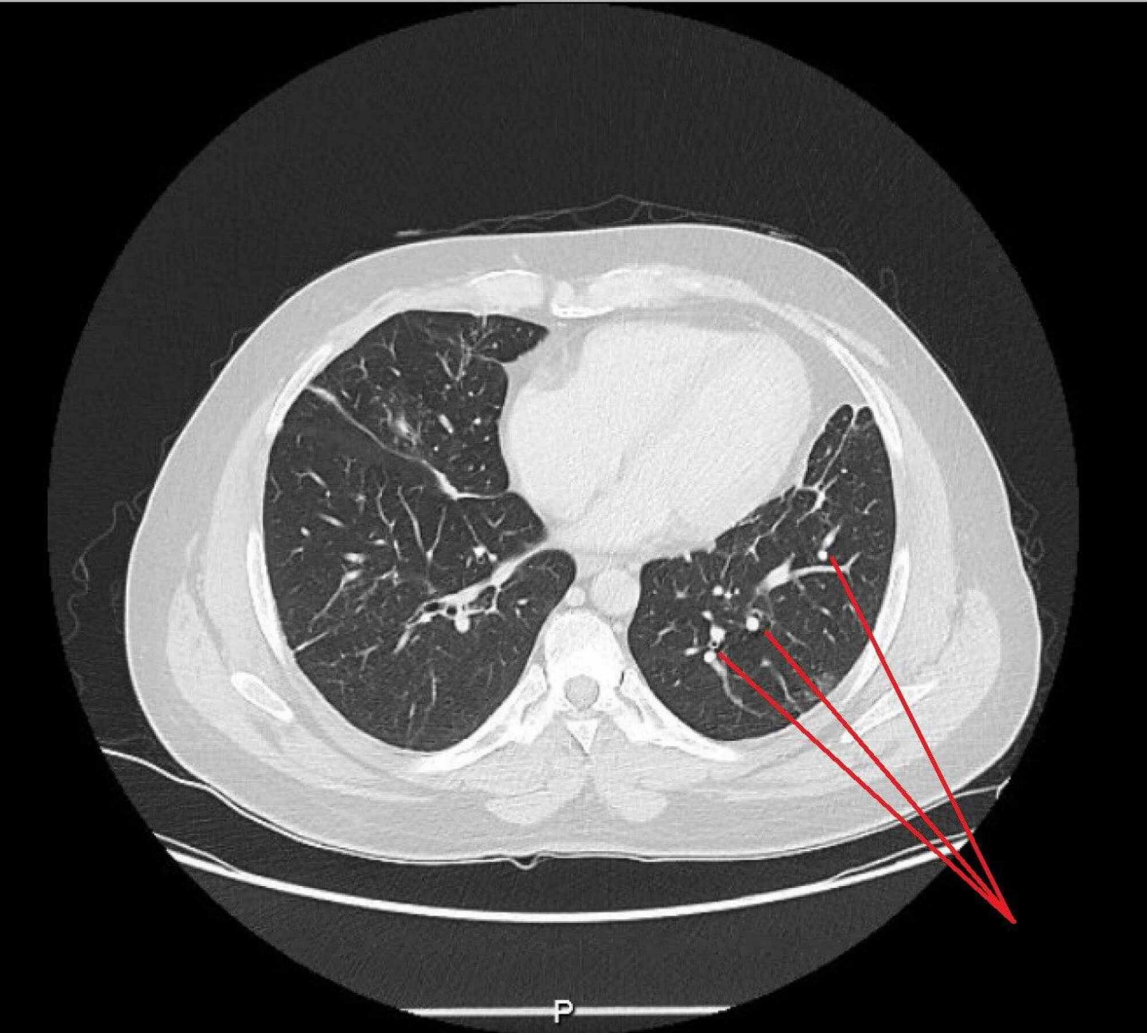
Computed tomography of the lungs showing micronodules

**Figure 3:**
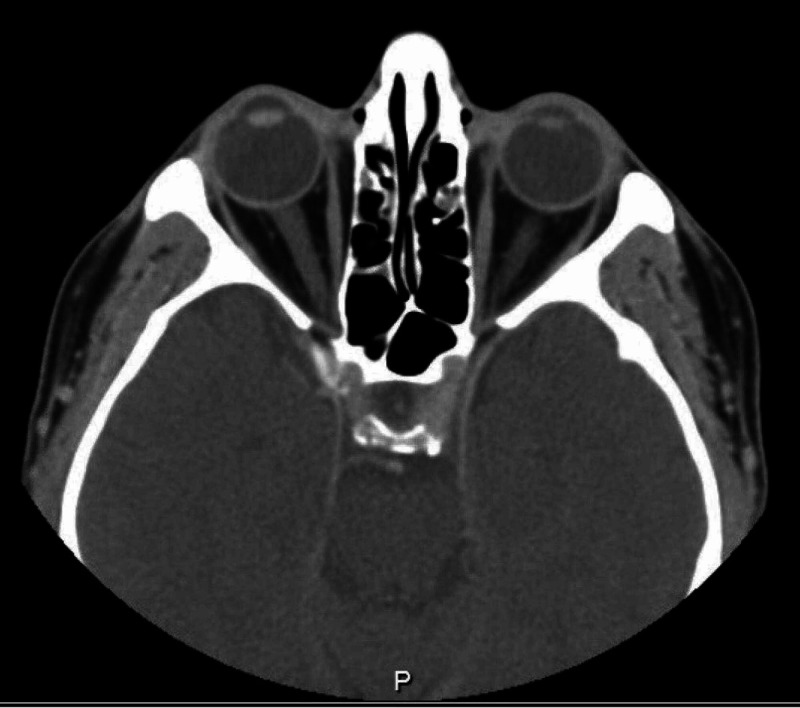
Brain and orbit computed tomography scan showing no abnormality in the orbital regions

Meanwhile, his chest CT scan reported stable left upper lobe fibrotic changes in association with volume loss and traction bronchiectasis with small atelectatic bands in both lower lobes. In addition, there were stable pulmonary micronodules in the right lower lobe with no obvious cavitary lesions or lung masses (Figure 2). Lastly, his PNS CT demonstrated bilateral otomastoiditis with interval development of severe left maxillary sinus mucosal disease and mild bilateral mucosal disease in the ethmoidal air cells bilaterally (Figure 3).

The initial plan for the patient was to start on 480 mg q8 hour trimethoprim/sulfamethoxazole (Bactrim) for one year for the Nocardiosis and to continue on a rituximab dose of 1 gram. His hospital course was uneventful, and the patient was advised to continue the treatment for Nocardia for a complete year with the recommendation to remove the dacryocystorhinostomy stent, CT follow-up, and regular culturing to confirm the eradication of the organism. Lastly, his last visit was to the rheumatology clinic in May 2020 and was in stable condition, with no more eye discharges, and, apparently, he improved with trimethoprim/sulfamethoxazole. From the rheumatology point of view, he was advised to continue on rituximab 1 gram every nine months. Meanwhile, he neglected all his visits to the ophthalmology and infectious disease clinics for swab and monitoring; after six months of continuous compliance with Bactrim, he stopped the medication by himself.

## Discussion

Nocardia is considered a very rare infectious opportunistic organism that is found in various sources of soil, with higher rates of organisms present in South Asia [6]. However, due to the continuous rise in infections, particularly in immunocompromised as well as chemotherapy patients, more organism species of Nocardia, which were not known to be infectious, are being reported in the literature. Nocardia cyriacigeorgica has been first described in 2001 by Yassin et al. [7] and is considered one of the emerging infections. In 2005, Thailand and Japan reported various incidences of Nocardia cyriacigeorgica as cases of brain abscess [8]. Meanwhile, in 2010, a sample of oil‐polluted sand samples collected from the Saudi Arabian desert showed contamination with Nocardia cyriacigeorgica [9]. A retrospective chart review in Saudi Arabia over a period of 16 years reported 19 patients with nocardiosis, with the main causative culprit being post-transplantation immunosuppression [10]. In addition, three types of Nocardia infections were reported: Nocardia asteroids, Nocardia brasiliensis, and Nocardia otitidiscaviarum, with the lung being the most common site of infection [10].

Around 52% of GPA patients can present with ophthalmic manifestation, such as scleral, episcleral, nasolacrimal, and corneal [11]. Early recognition of the cause of the ophthalmic manifestation and possible pathologies can be lifesaving and will help preserve eyesight from being lost in GPA patients. GPA can have a long list of various infectious manifestations, including but not limited to dermatological [12], cardiac [13], and nasal [14]. Not only that, the Nocardia infectious course of treatment are often exhaustive, with a long treatment duration that can reach up to a year, with the possibility of resistance to many antimicrobial agents such as trimethoprim-sulfamethoxazole [4] while long-term antimicrobial therapy is necessary to prevent a relapse infection of nocardiosis [15].

## Conclusions

GPA can be extremely unpredictable, with the possibility of being infected with various species of organisms that are not known to infect humans. This should elicit educating patients with the disease to always be cautious of their surroundings, avoid animal contact as much as possible, and carry on a more hygienic lifestyle than the normal individual. High suspicion of rare infections should be kept in mind while interacting with immunocompromised patients presenting with mild symptoms of infections.
